# New Data Define the Molecular Phylogeny and Taxonomy of Four Freshwater Suctorian Ciliates With Redefinition of Two Families Heliophryidae and Cyclophryidae (Ciliophora, Phyllopharyngea, Suctoria)

**DOI:** 10.3389/fmicb.2021.768724

**Published:** 2021-12-03

**Authors:** Mingzhen Ma, Yuqing Li, Qingxiang Yuan, Xuetong Zhao, Khaled A. S. Al-Rasheid, Jie Huang, Honggang Ma, Xiangrui Chen

**Affiliations:** ^1^School of Marine Sciences, Ningbo University, Ningbo, China; ^2^Institute of Evolution and Marine Biodiversity, Ocean University of China, Qingdao, China; ^3^Zoology Department, College of Science, King Saud University, Riyadh, Saudi Arabia; ^4^Key Laboratory of Aquatic Biodiversity and Conservation of Chinese Academy of Sciences, Institute of Hydrobiology, Chinese Academy of Sciences, Wuhan, China

**Keywords:** suctoria, taxonomy, molecular phylogeny, morphology, subtropical

## Abstract

Four suctorian ciliates, *Cyclophrya magna* Gönnert, 1935, *Peridiscophrya florea* (Kormos & Kormos, 1958) Dovgal, 2002, *Heliophrya rotunda* (Hentschel, 1916) Matthes, 1954 and *Dendrosoma radians* Ehrenberg, 1838, were collected from a freshwater lake in Ningbo, China. The morphological redescription and molecular phylogenetic analyses of these ciliates were investigated. Phylogenetic analyses inferred from SSU rDNA sequences show that all three suctorian orders, Endogenida, Evaginogenida, and Exogenida, are monophyletic and that the latter two clusters as sister clades. The newly sequenced *P. florea* forms sister branches with *C. magna*, while sequences of *D. radians* group with those from *H. rotunda* within Endogenida. The family Heliophryidae, which is comprised of only two genera, *Heliophrya* and *Cyclophrya*, was previously assigned to Evaginogenida. There is now sufficient evidence, however, that the type genus *Heliophrya* reproduces by endogenous budding, which corresponds to the definitive feature of Endogenida. In line with this and with the support of molecular phylogenetic analyses, we therefore transfer the family Heliophryidae with the type genus *Heliophrya* to Endogenida. The other genus, *Cyclophrya*, still remains in Evaginogenida because of its evaginative budding. Therefore, combined with morphological and phylogenetic analysis, Cyclophyidae are reactivated, and it belongs to Evaginogenida.

## Introduction

Ciliates are complex and well-developed single-celled eukaryotes which are mainly characterized by having cilia in their life history (Corliss, 1979; Lynn, 2008; Zhang et al., 2020). Ciliates have been studied for over three centuries, and estimates of the number of free-living ciliate species vary from three thousand to thirty thousand (Finlay et al., 1996; Foissner et al., 1999). The fact that ciliates are highly diverse and omnivorous means that they are considered to be a major link in the microbial food web and to play an important role in energy flow and material circulation in aquatic environments (Chi et al., 2020; Wu et al., 2020).

The subclass Suctoria Claparede & Lachmann, 1858, is a special group of ciliates. While the asexual reproduction of most ciliates is achieved by binary fission, the reproduction mode of suctorians is budding. This means that suctorians are polymorphic, with two distinct stages in their life history. Specifically, the sessile trophonts are usually non-ciliated but possess tentacles, while the free-swimming swarmers (larval forms) are typically ciliated (Chen et al., 2005, 2008a,b; Lynn, 2008; Song et al., 2009; Hu et al., 2019). Suctoria is divided into three orders based on their different modes of budding: Exogenida Collin, 1912, Endogenida Collin, 1912, and Evaginogenida Jankowski, 1978. This classification system is widely accepted by researchers, although some other more complex classifications have been proposed by protozoologists (Kormos and Kormos, 1958; Dovgal, 2002). There are about 560 suctorian ciliates widely distributed in various environments, such as marine, freshwater, and soil, as well as in the digestive tract of other organisms, as ectosymbionts on diverse invertebrates, or sometimes as endocommensals in hosts (Matthes, 1988; Foissner et al., 1999, 2002; Chen et al., 2008a,b; Marіño-Pérez et al., 2011; Hu et al., 2019). Most free-living suctorians are carnivorous, feeding primarily on other ciliates and flagellates, and thus, they are important components of the microbiological food web as predators (Lynn, 2008).

The characteristics of suctorian ciliates are mainly summarized as follows: (1) Body shapes are highly variable, from simple spheroid to flattened discs to complex branching forms; (2) tentacles are highly diverse, including prehensile, clavate, rod-like, and branched tentacles, which may be clustered in fascicles or scattered across the whole cell surface; (3) a lorica may be present or absent; (4) stalks are non-contractile, including both a real stalk and a stylotheca protuberance which is an extension of the posterior end of lorica; and (5) swarmer shape and infraciliature are important features for the identification of suctorians. Due to their highly diversified morphology and the fact that silver staining methods cannot be widely used for suctorians, there have historically been a mass of confusions and errors in the literature on suctorians. In recent years, however, research into suctorians is modernizing, and the more extensive application of staining, electron microscope, and molecular methods to their study has increased the availability of infraciliature, ultrastructure, and multi-gene sequence information. As a result, the taxonomic standard of suctorian ciliates is gradually improving (Batisse, 1994; Foissner et al., 1995; Chen et al., 2008a,b; Marіño-Pérez et al., 2011; Zhao et al., 2014).

In the present study, four suctorian ciliates, *Cyclophrya magna* Gönnert, 1935, *Peridiscophrya florea* (Kormos & Kormos, 1958) Dovgal, 2002, *Heliophrya rotunda* (Hentschel, 1916) Matthes, 1954, and *Dendrosoma radians* Ehrenberg, 1838, were isolated from a freshwater lake in Ningbo, China. They were investigated both *in vivo* and by using staining methods. Molecular data were reported for the first time for the latter three species, and the phylogenetic relationships within Suctoria were also analyzed based on SSU rDNA sequences.

## Materials and Methods

### Sample Collection, Observation, and Identification

**Figure 1:**
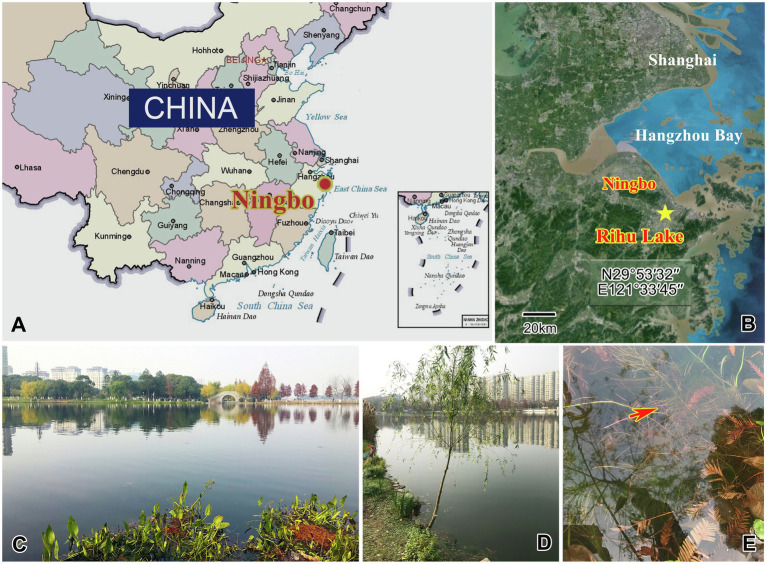
Maps and photographs of the sample site. **(A)** Location of Ningbo. **(B)** The star indicates the location of the sample site (29°53ʹ32"N, 121°33ʹ45"E). **(C)** Landscape of Rihu lake. **(D)** Suctorians were isolated from the root of this willow. **(E)** The red arrow showing the fibril of the willow.

Four species were collected from a subtropical freshwater lake, Rihu Lake (N29°53′32′′; E121°33′45′′), in Ningbo, China (Figure 1). *C. magna* is relatively common in summer when the water temperature is about 25°C. It was collected using artificial substrates (glass slides) which were immersed in water at a depth 0.5–1.0m for 7 to 15days during June 2016. *Peridiscophrya florea* was separated from the surface of fresh willow roots (*Salix babylonica*) immersed in water during May 2016 when the water temperature was about 20°C. *Heliophrya rotunda* was also collected using artificial substrates in January 2017 when the water temperature was about 9°C. *D. radians* was separated from the immersed surface of water hyacinth (*Eichhornia crassipes*) in February 2017 when the water temperature was about 11°C. All collected ciliates died within one or 2 days when maintained with habitat water at room conditions, regardless of being cultured with ciliates *in situ* or *Paramecium* sp. Thus, we could not culture either suctorian ciliate. It was possible, however, to separate enough individuals of the four species for morphological and molecular research.

Observations on living cells were undertaken using bright field and differential interference microscopy and measured at 100× to 1000× magnifications. The macronucleus shape and ciliary pattern of swarmers were revealed using the protargol impregnation method (Wilbert, 1975) or Methyl Green-Pyronin Staining (Beyotime, C0119, China). Terminology and systematics are mainly according to Matthes (1988), Dovgal (2002), Chen et al. (2008a), Chen et al. (2008b), and Lynn (2008).

### DNA Extraction, PCR Amplification, and Gene Sequencing

For every species, cells were optically identified and peeled off the substrates using an anatomic needle. Single cells were isolated and washed four times in ultra-pure water and then placed in 1.5-ml microfuge tubes with 45μl of buffer. Genomic DNA was extracted with the Dneasy Blood and Tissue Kit (Qiagen, Hilden, Germany) following the manufacturer’s instructions. Polymerase chain reaction (PCR) amplification of the SSU rDNA was performed using Q5^®^Hot Start High-Fidelity DNA Polymerase (NEB Co., Ltd., M0493, Beijing) with the universal eukaryotic primers 82F (5′ GAA ACT GCG AAT GGC TC 3′) and 18S-R (5′ TGA TCC TTC TGC AGG TTC ACC TAC 3′) (Medlin et al., 1988). An E.Z.N.A.^™^Quik Gel Extraction Kit (OMEGA Bio-Tek, D2500–01, Guangzhou) was used to purify PCR products, and a pEASY^®^–T1 Cloning kit (TransGen, CB101, Beijing, China) was used for cloning. Sequencing was performed bidirectionally (BGI Co., Ltd., Shanghai, China).

### Phylogenetic Analyses

The newly characterized SSU rDNA sequences, and the sequences of another 39 species/populations obtained from the NCBI GenBank database, were used for phylogenetic analyses. Although *C. magna* (AY007445, AY007446, AY007447, AY007448, and AY007449) is referred to as *Heliophrya erhardi* in the NCBI database, *H. erhardi* is a synonym for *C. magna*, and therefore, the latter name was used in the phylogenetic analyses reported here. Sequences were aligned using the GUIDANCE algorithm (Penn et al., 2010a) with MUSCLE parameters in the GUIDANCE web server (Penn et al., 2010b). Ambiguously aligned sites were refined using Gblocks v.0.91b (Castresana, 2000), and ambiguous columns were removed based on confidence scores calculated by GUIDANCE. Bayesian inference (BI) and maximum likelihood (ML) analyses were carried out online on the CIPRES Science Gateway v 3.3).^1^ The best fitting model for phylogenetic analyses was selected by MrModeltest v2.2 (Nylander, 2004). Bayesian inference (BI) analysis was performed with MrBayes 3.2.6 (Ronquist et al., 2012) using the GTR+I+G evolutionary model (Nylander, 2004). The program was run for 1,000,000 generations with a sample frequency of 100, and a burn-in of 2500. ML analysis was performed with RAxML-HPC2 on XSEDE v8 (Stamatakis, 2014) using the GTR+I+G model as selected by Modeltest v3.4 (Posada and Crandall, 1998). The reliability of the ML internal branches was assessed using a nonparametric bootstrap method with 1000 replicates. MEGA v5.0 (Tamura et al., 2011) was used to visualize tree topologies.

## Results

### Zoobank Registration

**Class Phyllopharyngea de Puytorac et al., 1974**

**Subclass Suctoria Claparède & Lachmann, 1858**

**Order Evaginogenida Jankowski, 1978**

**Family Cyclophryidae Jankowski, 2007**

**Genus *Cyclophrya* Gönnert, 1935**

**Species *Cyclophrya magna* Gönnert, 1935**

*Cyclophrya magna* is mainly characterized by the disc-like body and multiple tentacles in fascicles. This well-known species has been reported several times in the half century since the original description. Most of these reports, however, focused on the ultrastructure of the tentacles, rather than attempting to present accurate morphological data and taxonomic research (Hauser and Eys, 1976; Spoon et al., 1976; Hanke-Bücker et al., 2000). There is therefore a need to provide an improved diagnosis here.

### Improved Diagnosis

Disc-like body about 50–190μm in diameter, some of them are oval. Transparent adhesive disc obvious, with a ring width of about 3–12μm. Capitate tentacles clustered in numerous fascicles along the body margin, mostly in 3–9 fascicles. Contractile vacuoles about 3–14. Macronucleus branched.

### Morphological Description of Ningbo Population

**Figure 2:**
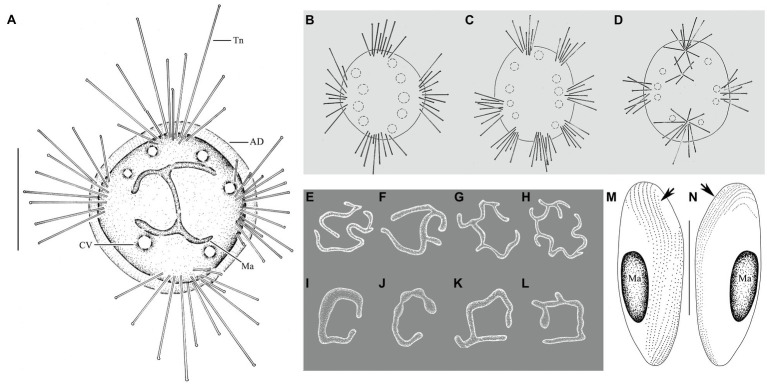
*Cyclophrya magna* Gönnert, 1935 from life **(A–D)** and after protargol impregnation **(E–L)** as well as its swarmer after protargol impregnation **(M,N)**. **(A)** *Cyclophrya magna in vivo*. **(B–D)** Different number of tentacle fascicles and contractile vacuoles. (E–L) Different shapes of macronuclei. **(M,N)** Views of swarmer, arrows to show somatic kineties. AD, adhesive disc; CV, contractile vacuole; Ma, macronucleus; Tn, tentacle. Scale bars=50μm.

**Figure 3:**
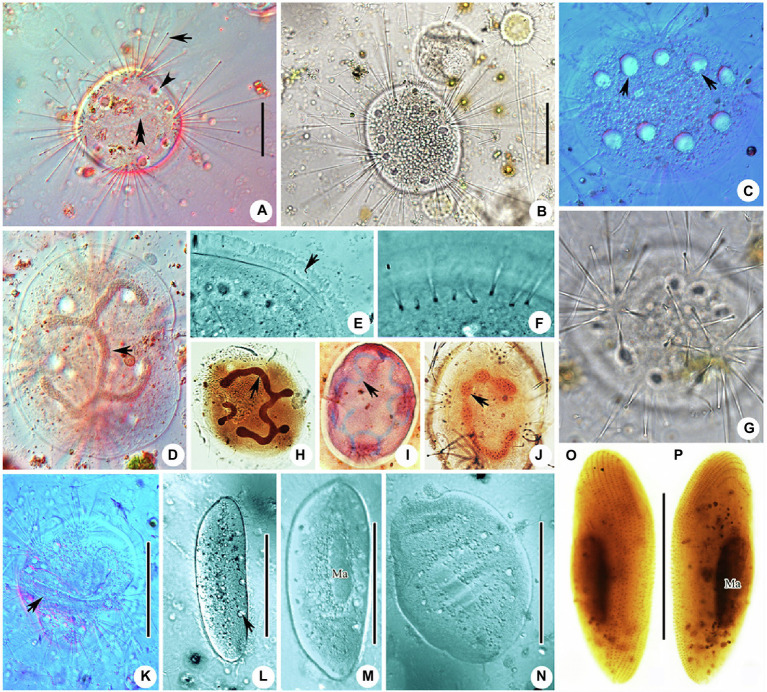
Photomicrographs of *Cyclophrya magna* Gönnert, 1935 and its swarmer from life **(A–G,K–N)** and after protargol staining **(H,J,O,P)** as well as Methyl Green-Pyronin Staining **(I)**. **(A)** *Cyclophrya magna in vivo*. Arrow to show the tentacles. Arrowhead to show contractile vacuole. Double arrowheads to show macronucleus. **(B–D,G)** To show the different individuals with different number of tentacle fascicles and contractile vacuoles. Arrows in **(C)** show the contractile vacuoles. Arrow in **(D)** to indicate macronucleus *in vivo*. **(E)** The part of living trophont. Arrow protruding adhesive disc (ring). **(F)** To show tentacle fascicle. Some tentacles arranged in rows. **(H–J)** After protargol staining **(H,J)** and Methyl Green-Pyronin Staining **(I)**, arrows to show different shapes of macronucleus. (K) View of an individual during reproduction, arrow to indicate the swarmer. **(L–N)** The process from swarmer growing into trophont. Arrow in **(L)** to indicate contractile vacuole. **(O,P)** View of swarmer after protargol staining. Ma, macronucleus. Scale bars=50μm.

Trophont body flat disc-shaped, without lorica or stalk, directly attached to substrates using a transparent adhesive disc (Figures 2A, 3A,E). Body size 70–160μm×60–160μm *in vivo*, usually about 90μm×100μm, adhesive disc width about 3–11μm. Capitate tentacles straight and clustered in 3–9 fascicles (usually in four fascicles; Table 1), each fascicle including 3–20 tentacles. When fully extended, tentacles up to 260μm in length (Figures 2B–D, 3A–C,G). Tentacles sometimes arranged in a line in one fascicle (Figure 3F). Contractile vacuoles usually distributed around body margin, sometimes arranged in approximately two parallel lines (Figures 2B–D, 3C). Highly variable number of contractile vacuoles, about 4–14. Macronucleus filiform and irregularly branched, usually concentrated in the middle of body (Figures 2E–L, 3D,H–J).

**Table 1:** Morphological characterization of four suctorians.

Characters	Species	Min	Max	Mean	M	SD	CV	n
Body length	*C. magna*	70	160	96.8	85	25.3	26.1	25
	*P. florea*	55	200	122.9	118	30.2	24.6	25
	*H. rotunda*	40	80	61.2	60	8.2	13.4	27
	*D. radians*	150	2270	1022.9	906	584.6	57.2	20
Body width	*C. magna*	60	160	89.8	80	22.7	25.3	25
	*P. florea*	15	75	42.0	40	11.2	26.7	25
	*D. rotunda*	5	70	27.2	20	18.8	69.1	20
No. tentacle fascicle	*C. magna*	3	9	4.3	4	1.3	30.2	25
	*P. florea*	1	1	1.0	1	0	0	25
	*H. rotunda*	8	22	13.8	14	2.9	21.0	27
	*D. radians*	1	29	10.3	8	7.6	73.8	20
Extended length of tentacle	*C. magna*	65	260	111.0	105	43.9	39.5	25
	*P. florea*	45	200	120.5	110	39.5	32.8	22
	*H. rotunda*	25	190	69.3	55	35.1	50.6	23
	*D. radians*	50	250	133.4	125	51.5	38.6	20
No. of contractile vacuole	*C. magna*	4	14	6.7	6	2.1	31.3	25
	*P. florea*	1	4	2.1	2	1.2	57.1	15
	*H. rotunda*	5	22	11.5	11	3.8	33.0	26
	*D. radians*	4	70	26.9	22	18.6	69.1	18
stylotheca length	*P. florea*	40	250	126.3	120	67.5	53.4	25
stylotheca width	*P. florea*	8	20	16.8	15	3.8	22.6	25
Macronuclear length	*H. rotunda*	14	28	20.9	21	3.9	18.7	27
Macronuclear width	*H. rotunda*	10	19	13.8	14	2.6	18.8	27

Macronuclear length and width are based on protargol-stained specimens, and all other data are based on morphology in vivo. Morphometric data of them are based on Ningbo population. Measurements in μm. CV, coefficient of variation in %; Max, maximum; Mean, arithmetic mean; Min, minimum; n, number of cells measured; SD, standard deviation; SE, standard error of arithmetic mean.

Swarmer formed by evaginative pattern (Figure 3K). Newly born swarmer swimming freely in water, slender ellipsoid, or finger-shaped, about 100μm×35μm *in vivo*, 80μm×30μm after protargol impregnation. Body surface densely covered with cilia and arranged in nine longitudinal ciliary rows. (Figures 2M,N, 3O,P). Contractile vacuoles about 10–14, arranged in two lines. Macronucleus ellipsoidal or rod-shaped, about 30μm×15μm (Figures 2M,N, 3O,P). Swarmer develops into adult quickly; free-swimming individuals attach to substrates and expand into a flat disc-like shape within several minutes (Figures 3L–N).

**Family Periacinetidae Jankowski, 1978**

**Genus *Peridiscophrya* Nozawa, 1938**

**Species *Peridiscophrya florea* (Kormos & Kormos, 1958) Dovgal, 2002**

The taxonomic position of *Peridiscophrya florea* has been changed many times since its first discovery, and an accurate morphological description is also still lacking. It was discovered in Hungary and reported by Kormos and Kormos (1958) who described the type species of their new genus *Catharina*, named *Catharina florea*. Two years later, *Catharina* was substituted as *Caracatharina* Kormos, 1960 in Corliss (1960). Matthes (1988) transferred it to *Discophrya* Claparede & Lachmann, 1859. Dovgal (2002) moved it to *Peridiscophrya* without any description or illustration. Zharikov et al. (2005) only supplied a short redescription based on a Russian population. Thus, an improved diagnosis is needed here based on detailed morphological characters.

### Improved Diagnosis

Ellipsoidal or finger-shaped body enclosed in the distal region cup-like lorica, about 55–200μm×15–75μm *in vivo*. Lorica colorless and transparent, apical cup-shaped, antapical prolonged like a stalk (stylotheca), about 1–2.5 times length of upper part. About 100–200 capitated tentacles concentrated on the apical surface of the body, up to 200μm in length. Contractile vacuoles present, about 1–4 in number. Macronucleus filiform, sometimes with short branches.

### Morphological Description of Ningbo Population

**Figure 4:**
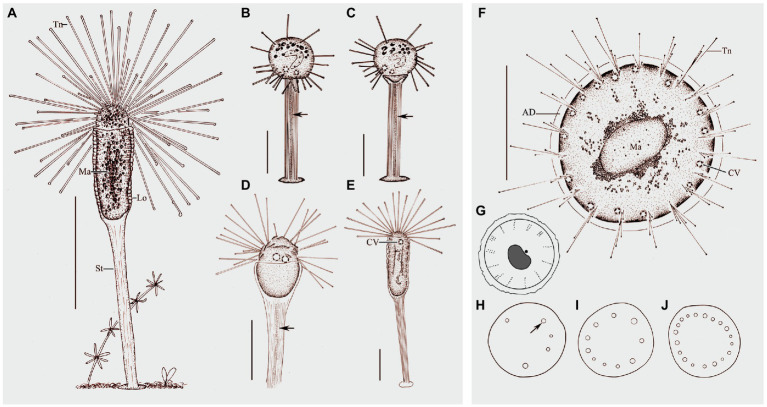
Morphology of *Peridiscophrya florea* (Kormos & Kormos, 1958) Dovgal, 2002 **(A–E)** and *Heliophrya rotunda* (Hentschel, 1916) Matthes, 1954 **(F–J)**. **(A)** View of *Peridiscophrya florea* from life. **(B–E)** The growth process of *P. florea*. Arrow marks some particles in stylotheca. **(F)** *Heliophrya rotunda in vivo*. **(G)** Arrangement of tentacles. **(H–J)** Different individuals with different number of contractile vacuoles. Arrow marks the contractile vacuole. AD, adhesive disc; CV, contractile vacuoles; Lo, lorica; Ma, macronucleus; St, stalk; Tn, tentacle. Scale bars=100μm (A), = 50μm **(B–F)**.

**Figure 5:**
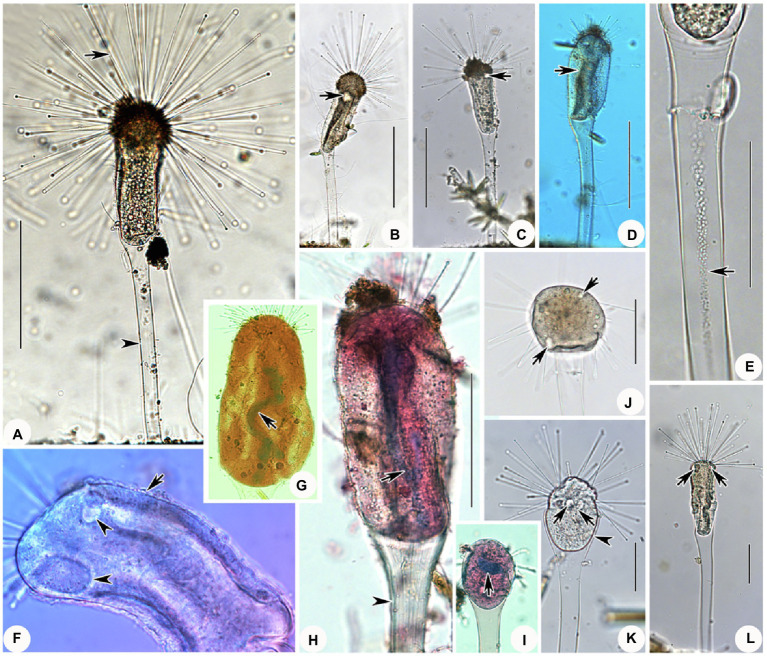
Photomicrographs of *Peridiscophrya florea* (Kormos & Kormos, 1958) Dovgal, 2002 from life **(A–F**,**J–L)** and after Methyl Green-Pyronin Staining **(G–I)**. **(A)** *Peridiscophrya florea in vivo*. Arrow to show tentacle. Arrowhead to show stylotheca. **(B–D)** Different individuals. Arrows in **(B,C)** to indicate contractile vacuoles. Arrow in **(D)** to indicate longitudinal furrows. **(E)** Same individual as **(J–L)** enlarged to show the particles (arrow) in stylotheca. **(F)** Enlarged the ciliate body. Arrow to show lorica with dense rings. Arrowheads to show the contractile vacuoles. **(G–I)** *Peridiscophrya florea* after Methyl Green-Pyronin Staining. Arrow in **(G)** to show the filiform macronucleus. Arrow in **(H)** indicates ramified macronucleus. Arrowhead to show vertical lines on the stylotheca. Arrow in **(I)** to show rod-like macronucleus during the growth process. **(J–L)** The process of lorica growth. Arrows in these pictures to show contractile vacuoles. Arrowhead in **(K)** to show lorica. Scale bars=100μm **(A–D)**, = 50μm **(E**, **H**,**J–L)**.

Cell body ellipsoidal or finger-shaped, about 55–200μm×15–75μm in size, usually about 120μm×40μm (Table 1). Cytoplasm colorless to lightly brownish. Sessile trophont almost enclosed by transparent lorica and attached to substrates by means of stylotheca posterior end of lorica (Figures 4A, 5A). Lorica symmetrical, champagne glass-shaped, 60–210μm×20–80μm, upper part always with 8–15 transverse stripes, thin or hungry individual with four longitudinal furrows; lower part of lorica prolonged and forming a stylotheca, about 40–250μm×8–20μm, sculptured with inconspicuous longitudinal lines (Figures 4A, 5A–D), end of lorica equipped with adhesive disc (Figures 4B,C). Capitate tentacles about 100–200 with different length, longest ones about 200μm long, and all the tentacles confined on the apical region of cell body. Contractile vacuoles about 1–4 in number, usually positioned near the apical region (Figures 4A,E, 5F,L). Macronucleus filiform, sometimes with one or two branches (Figures 4A,E, 5G,H). Micronucleus not observed.

Free-swimming swarmer not observed. Newly attached individual spherical, exposed at the top of lorica, cytoplasm colorless with many granular inclusions scattered in upper part of cell. Capitate tentacles scattered across the whole cell surface (Figures 4B,C, 5J). Transparent stylotheca, without cup-shaped structure, with longitudinal stripes on the surface and many gray particles arranged in a line along the inner axis (particles absent in trophont individuals, function unknown; Figures 4B–D, 5E), terminal adhesive disc obvious. Macronucleus curved sausage shape (Figure 5I). Contractile vacuoles not confined to apical region (Figures 4B,C, 5J,K). Subsequently, upper part of lorica gradually expanded to form a cocktail glass-shaped structure (Figures 4D, 5K). Then, lorica cup growing bigger and longer, gradually enclosing cell body which is compressed to become ellipsoidal or finger-shaped (Figures 4E, 5L), contractile vacuoles and tentacles also moved to apical region (Figures 4E, 5L).

**Order Endogenida Collin, 1912**

**Family Heliophryidae Corliss, 1979**

**Genus *Heliophrya* Saedeleer & Tellier, 1930**

**Species *Heliophrya rotunda* (Hentschel, 1916) Matthes, 1954**

*Heliophrya rotunda* is a common freshwater species which has been found several times since its discovery (Hentschel, 1916; Saedeleer and Tellier, 1930; Gönnert, 1935; Rieder, 1936, 1988; Matthes, 1954, 1988; Mogensen and Butler, 1982; Foissner et al., 1995). A clear definition has never been provided, however, and its classification is still controversial. Accurate taxonomic identification and improved diagnosis are therefore needed based on well characterized morphological features.

### Improved Diagnosis

Body shape flat-disc, diameter about 30–90μm *in vivo*. Transparent adhesive disc about 6μm in width. Capitate tentacles up to 190μm long and arranged in many fascicles along cell margin. About 3–22 contractile vacuoles. Single macronucleus, oval or kidney-shaped.

### Morphological Description of Ningbo Population

**Figure 6:**
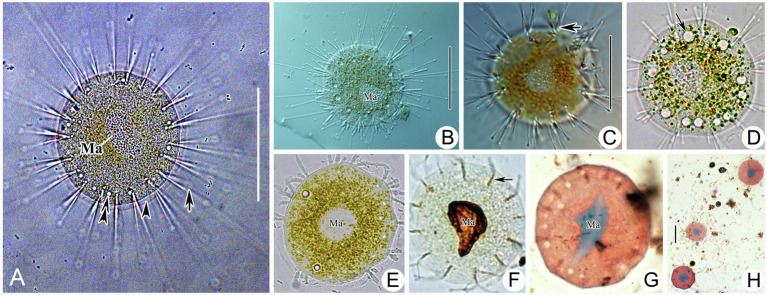
Photomicrographs of *Heliophrya rotunda* (Hentschel, 1916) Matthes, 1954 from life **(A–E)** and after protargol staining **(F)** as well as Methyl Green-Pyronin Staining **(G,H)**. A. *Heliophrya rotunda in vivo*. Arrow to show tentacle. Arrowhead to show adhesive disc. Double arrowheads to show contractile vacuole. **(B–E)** To show different individuals. Arrow in **(C)** to indicate tentacles in rows. Arrow in **(D)** to indicate contractile vacuole. **(F)** *Heliophrya rotunda* after protargol staining. Arrow to show the tentacle arrangement, to indicate tentacles in rows. **(G,H)** *Heliophrya rotunda* after Methyl Green-Pyronin Staining. Ma, macronucleus. Scale bars=50μm.

Stalkless body flat disc-shaped, without lorica, attached to the substrates by transparent adhesive disc (Figures 4F, 6A). Body diameter 40–80μm *in vivo*, usually about 60μm (Table 1). Adhesive disc transparent, 4–6μm in width, easily detected (Figures 4F, 6A,E; Table 1). Capitate tentacles slim and with high contractility, extended tentacles up to 190μm long, contracted ones like springs. Most tentacles inclined upwards and held at an angle of up to 90° to cell surface. All fascicles composed of about 8–22 tentacles along cell margin. Each fascicle including about eight tentacles arranged in 1–3 radial rows (Figures 4G, 6C,D,F). About 5–22 contractile vacuoles with different sizes, distributed around body border (Figures 4H–J, 6A,D). Cytoplasm colorless but usually greenish or brownish due to mass of green granular inclusions (Figures 6B–E). Macronucleus shape slightly variable, usually oval, sometimes kidney-shaped, always located in the center of cell (Figures 6E–H).

**Family: Dendrosomatidae Fraipont, 1878**

**Genus: *Dendrosoma* Ehrenberg, 1838**

**Species *Dendrosoma radians* Ehrenberg, 1838**

*Dendrosoma radians* is a well-known suctorian ciliate with a ramified body. Since the original report by Ehrenberg (1838), it has been reinvestigated many times in the last two centuries (Kent, 1882; Hickson and Wadsworth, 1909; Penard, 1920; Gönnert, 1935; Bick, 1972; Matthes, 1988; Foissner et al., 1995; Dovgal, 1996). Here, we combine all the historic descriptions and present data to provide an improved diagnosis.

### Improved Diagnosis

Ramified body in the form of an individual tree or colony. Huge differences in cell size, from 150 to 5000μm in height, 5–70μm in width of stem. Each end of branch with one fascicle of capitate tentacles, fully extended tentacles about 250μm in length. Contractile vacuoles numerous, randomly distributed along the stem and branches. Macronucleus filiform and expanded in stem and branches. Swarmer ellipsoid.

### Description of Ningbo Population

**Figure 7:**
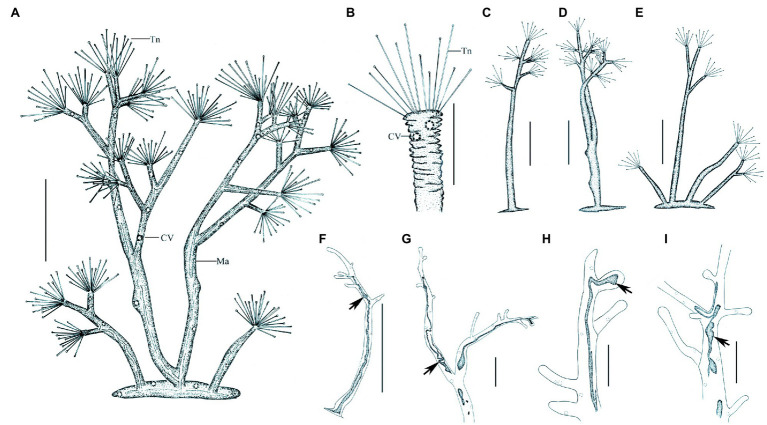
Morphology of *Dendrosoma radians* Ehrenberg, 1838 from life **(A–E)** and after protargol staining **(F–I)**. **(A)** View of a typical individual. **(B)** Part of branch, to show some ends of branches can shrink. **(C–E)** View of different individuals in different periods of life history. **(F–I)** After protargol staining and Methyl Green-Pyronin Staining, arrows to show the different macronucleus. CV, contractile vacuole; Ma, macronucleus; Tn, tentacle. Scale bars=250μm **(A,D–E)**, = 50μm **(B–C,G–I)**, = 500μm **(F)**.

**Figure 8:**
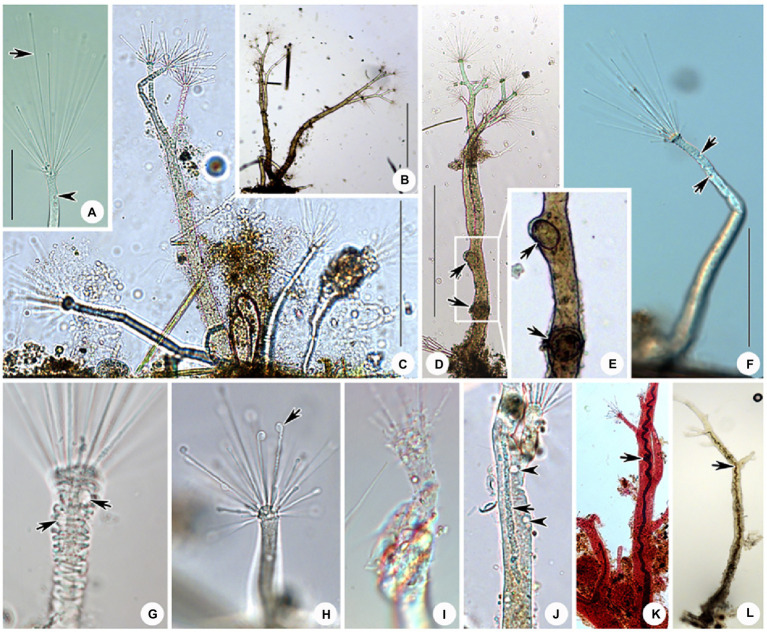
Photomicrographs of *D. radians* Ehrenberg, 1838 from life **(A–J)** and after protargol staining **(L)** as well as Methyl Green-Pyronin Staining **(K)**. **(A)** Part of branch, arrow to show the fully stretched tentacle, arrowhead to show contractile vacuole. **(B–D,F)** View of different individuals in different periods of life history. **(E)** Same individual as **(D)** enlarged to show the swarmers (arrows). Arrows in **(D,E)** to show the swarmers. Arrows in **(F)** to show contractile vacuoles. **(G)** Part of branch, to show some ends of branches can shrink. Arrows to show contractile vacuoles. **(H)** Arrow to show the spherical end of shrink tentacle. **(I)** Under unfavorable conditions tentacles and tentacle actinophores can be melted. **(J)** Arrow to show the filiform macronucleus, which traverses the whole cell. Arrowheads show the contractile vacuoles. **(K)** After Methyl Green-Pyronin Staining, arrow to show the filiform macronucleus. **(L)** After protargol staining, arrow to show the filiform macronucleus. Scale bars=50μm (A), = 500μm **(B–D,F)**.

Ramified body stalkless, without lorica, attached to substrates by basal body surface. Younger individuals colorless to light gray, straight or curved stick-shaped, not branched, usually about 200μm tall (Figures 7C, 8F). Developing individuals gray or brownish, main body with several branches, body size about 1,000μm (Figures 7D,E, 8B,D). Developed individuals brownish, ramified body composed of several stems and numerous branches, stems connected to each other by irregularly shaped baseplate; ramified body size up to 2,270μm tall (Figures 7A, 8C and Table 1). Distal portion of branch colorless and translucent, with slight contractility; contracted branch spring-like with many folds; end of branch flat or slightly protuberated, covered with numerous capitate tentacles (Figures 7B, 8A,G,H). Highly contractile capitate tentacles; fully extended ones about 50–250μm in length, contracted ones spring-like with expansion of spherical ends (Figure 8H). Under unfavorable conditions, younger individuals gradually melted and disappeared (Figure 8I). Contractile vacuoles numerous but highly variable in number (4–70), irregularly distributed along the body stem and branches, and even the irregular shaped baseplate (Figures 7A, 8J). Macronucleus filiform and interspersed in body stems and branches, including baseplate, usually in elongated belt, some parts broken or fused into expanded nodes (Figures 7F–L).

Swarmer formed by endogenous pattern, ellipsoid, about 50μm×30μm in size. New swarmers formed in main stems (Figures 8D,E).

### Molecular Data and Phylogenetic Analyses

#### Sequence Information

SSU rDNA sequences of four isolates were obtained in the present study and have been deposited in the GenBank database with the length (bp), GC content and GenBank accession numbers as follows: *Peridiscophrya florea* (1563bp, 44.72%, MZ912680), *C. magna* (2350bp, 44.30%, MZ912681), *Heliophrya rotunda* (2803bp, 45.13%, MZ912682), and *D. radians* (2018bp, 42.72%, MZ912683). The latter three species have introns.

#### Phylogenies Inferred From the SSU rDNA

**Figure 9:**
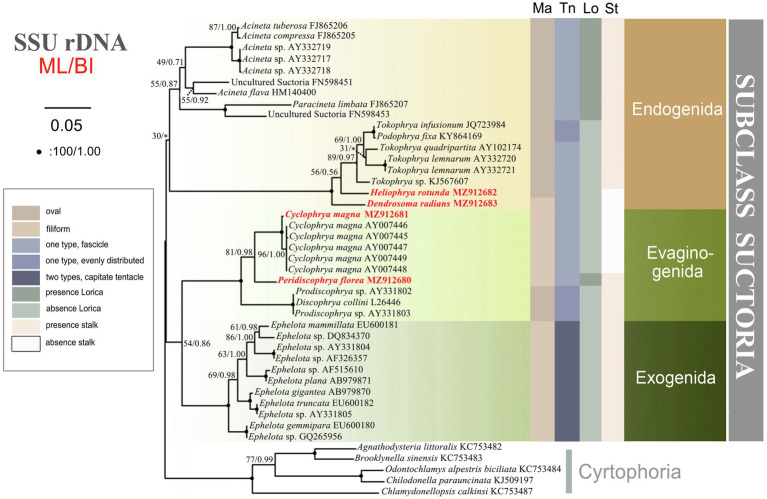
The consensus phylogenetic trees inferred from small subunit ribosomal DNA (SSU rDNA) using maximum likelihood and Bayesian analysis methods. Numbers at nodes represent the bootstrap values of ML and the posterior probabilities of Bayesian analysis (BI), respectively. ^*^the disagreement between BI tree and the reference ML tree. Fully supported (100%/1.00) branches are marked with solid circle. Sequences newly added in the present work are in red. The scale bar corresponds to 5 substitutions per 100 nucleotide positions.

The SSU rDNA-based tree was constructed as shown in Figure 9. Since the topologies of the ML and BI trees were basically concordant just the topology of the ML tree is presented with support values from both algorithms indicated on the branches. The analysis includes all species of Suctoria for which SSU rDNA sequence data are available and five species of Cyrtophoria as the outgroup. It reveals that all three orders within Suctoria are monophyletic; that Evaginogenida and Exogenida are sister taxa; that *Heliophrya rotunda* and *D. radians* fall within Endogenida; and that *C. magna* and *Peridiscophrya florea* cluster together in Evaginogenida.

## Discussion

### Redefinition of the Family Heliophryidae Corliss, 1979 and Cyclophryidae Jankowski, 2007

*Cyclophrya magna* was found by Gönnert (1935) and described as the type species of his newly established genus *Cyclophrya*. This genus was originally attributed to the family Dendrosomatidae Fraipont, 1878. At that time, Dendrosomatidae included another discoid genus, *Heliophrya* Saedeleer & Tellier, 1930. Most species in Dendrosomatidae, however, possess actinophores bearing tentacles. Since *Cyclophrya* and *Heliophrya* do not have these structures, Corliss (1979) established a new family for these two genera, Heliophryidae. Two years later, Jankowski put forward a point that *Solenophrya crassa* Claparede & Lachmann, 1859 is an older synonym for the name *C. magna*. Accordingly, he proposed to rename the family Heliophryidae Corliss, 1979 (which included the genus Cyclophrya) as Solenophryidae Jankowski, (1981). Seven years later, Rieder (1988) pointed out that *Cyclophrya* and *Heliophrya* exhibited evaginative and endogenous modes of budding, respectively, which suggested that the establishment of family Heliophryidae was incomplete and limited. He proposed establishing a new family for *Cyclophrya*, Cyclophryidae, and moving Heliophryidae to Endogenida. Rieder’s suggestion has not been widely accepted by subsequent protozoologists, however. Until 2007, Jankowski agreed with Rieder’s point of view and he transferred Heliophryidae from Evaginogenia to Endogenia. He established a new family Cyclophryidae for *Cyclophrya* in Evaginogenia. However, there was no evidence presented to support his claim. At the same time, he also considered *Solenophrya crassa* Claparede & Lachmann, 1859, *Craspedophrya erhardi* Rieder, 1936, *Heliophrya erhardi* (Rieder, 1936) Matthes, 1954 and *Trichophrya maxima* Oppenheim, 1957 are older synonyms for *Cyclophrya magna* Gönnert, 1935.

In the present study, we verify that the reproductive modes of *Heliophrya* and *Cyclophrya* are indeed as described in Rieder (1988); that is, the budding mode of *Heliophrya* is endogenous, while *Cyclophrya* is evaginative. Since within Suctoria, the reproductive mode is the sole basis for the classification of order taxa, Heliophryidae/*Heliophrya* must indeed be transferred from Evaginogenida to Endogenida, while the genus *Cyclophrya* remains in Evaginogenida and still in Cyclophryidae Jankowski, 2007. The present phylogenetic analyses support this morphologically based reassignment: that is, *Heliophrya* is completely aggregated in Endogenida, and *Cyclophrya* is aggregated in Evaginogenida. In other words, our research fully supports the establishment of Cyclophryidae Jankowski, 2007 based on morphological and molecular data.

**Figure 10:**
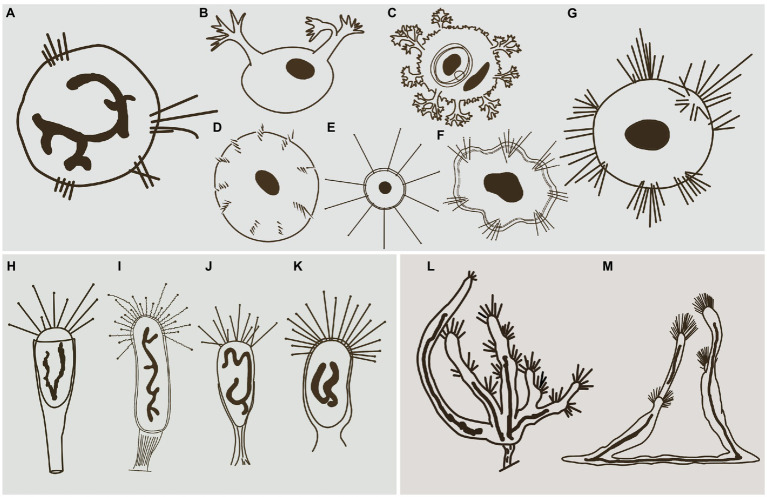
Some selected species that are morphologically similar to four species in this study. **(A)** *Cyclophrya magna* Gönnert, 1935 (after Dovgal, 2002). **(B)** *Dendrocometes paradoxus* Stein, 1851 (after Dovgal, 2002). **(C)** *Niscometes peregrinus* Small & Lynn, 1958 (after Dovgal, 2002). **(D)** *Discosomatella tenella* Swarczewsky, 1928 (after Dovgal, 2002). **(E)** *Heliophrya minima* (Rieder, 1936) Foissner, 1988 (after Foissner et al., 1995). **(F)** *Heliophrya sinuosa* Rieder, 1936 (after Rieder, 1936). **(G)** *Heliophrya rotunda* (Hentschel, 1916) Matthes, 1954 (after Dovgal, 2002). **(H)** *Peridiscophrya florea* (Kormos & Kormos, 1958) Dovgal, 2002 (after Zharikov et al., 2005). **(I)** *Discophrya robusta*, Nozawa, 1938 (after Nozawa, 1938). **(J)** *P. crassipes* (Rieder, 1936) Dovgal, 2002 (after Zharikov et al., 2005). **(K)** *Peridiscophrya japonica* Nozawa, 1938 (after Zharikov et al., 2005). **(L)** *Dendrosomides paguri* Collin, 1906 (after Dovgal, 2002). **(M)** *D. radians* Ehrenberg, 1838 (after Dovgal, 2002).

Hitherto, apart from *Cyclophrya* Gönnert, 1935 (Figure 10A) and *Heliophrya* Saedeleer & Tellier, 1930, three other genera possess a disc-shaped body, all of which belong to the order Evaginogenida, namely *Discosomatella* Corliss, 1960 (Figure 10D), *Dendrocometes* Stein, 1852 (Figure 10B), and *Niscometes* Jankowski, 1987 (Figure 10C). *Cyclophrya* is easily distinguished from *Discosomatella* by the arrangement of tentacles in each group (disordered clustering vs. arranged radially in a line; Dovgal, 2002). *Cyclophrya* differs from *Dendrocometes* and *Niscometes* in the shape of its tentacles (straight and capitate vs. branched and terminal tapering; Dovgal, 2002).

### *Cyclophrya magna* Gönnert, 1935

*Cyclophrya magna* was first reported by Gönnert in 1935. Matthes (1988) provided a short description of this species and considered two other species as synonymous with it; that is, *Craspedophrya erhardi* Rieder, 1960 and *H. erhardi* (Rieder, 1936) Matthes, 1954. Matthes (1954) mentioned that this species had both evaginative and endogenous types of budding, which did not meet the classification standard for Suctoria. Furthermore, Matthes (1988) showed that *C. magna* had between 1 and 14 tentacle fascicles, and the range is too wide for a suctorian species. It is therefore suspected that other species have been confused in Matthes’ description of *C. magna*. In Dovgal (2013), the main differences between *C. magna* and *C. katharinae* are arrangement of tentacles and the body size. It can be assumed that these are the manifestation of intraspecific variability. Therefore, *C. katharinae* is regarded as a junior synonym for *C. magna*. Hence*, Cyclophrya* is a monotypic genus.

### *Peridiscophrya florea* (Kormos & Kormos, 1958) Dovgal, 2002

*Peridiscophrya florea* was first reported as *Catharina florea* Kormos & Kormos, 1958. Two years later, it was transferred to *Caracatharina* Kormos, 1960. Matthes (1988) thought that *Caracatharina* was a synonym of *Discophrya* Claparede & Lachmann, 1859 and moved this species to *Discophrya*. Recently, Dovgal (2002) transferred it to *Peridiscophrya* Nozawa, 1938 which was mainly characterized by the cylindrical or finger-like body covered with lorica, a sturdy stylotheca, capitate tentacles in a single fascicle, and a ramified macronucleus. *Peridiscophrya* differs from *Discophrya* mainly in the stylotheca (Figures 10H,I). Based on these descriptions and comparisons, we agree with Dovgal’s classification of this species.

There are two other nominal species in this genus, that is, *Peridiscophrya japonica* Nozawa, 1938 (Figure 10K) and *P. crassipes* (Rieder, 1936) Dovgal, 2002 (Figure 10J). The type species, *P. japonica*, was collected by Nozawa from Kyoto, Japan, and differs from *P. florea* in three aspects of the lorica. Namely, in *P. japonica*, the lorica enveloped the whole cell body (vs. the anterior part of body leaked outside of the lorica), is smooth (vs. lorica horizontally striped in *P. florea*), and possesses a short and smooth stylotheca that extends up to one-third of the apical part of the lorica (vs. slender and vertically striped, about 1–2.5 times the upper part of lorica in *P. florea*).

*Peridiscophrya crassipes* was collected from a fishpond in Switzerland by Rieder (1936) (Figure 10J). It also differs from *P. florea* in the character of its lorica (covering one-third to half of body length vs. covering four-fifths or whole of body length), as well as in the location of its contractile vacuoles (end of the body vs. top of the body).

### *Heliophrya rotunda* (Hentschel, 1916) Matthes, 1954

*Heliophrya rotunda* was first reported by Hentschel (1916) and named as *Trichophrya rotunda*. This was an obvious misidentification, however, because this species lacks actinophores to support the fascicle of tentacles and thus should not be classified into the genus *Trichophrya*. Saedeleer and Tellier (1930) found a similar organism, but they missed Hentschel’s publication and described it as a new species, that is, *Heliophrya collini* Saedeleer & Tellier, 1930. Matthes (1954) combined *Trichophrya rotunda* with the genus *Heliophrya*, and treated *Heliophrya collini* as a synonym of *Heliophrya rotunda* (Hentschel, 1916) Matthes, 1954. In addition, Gönnert (1935) and Rieder (1936) each established *Platophrya* and *Craspedophrya* for *Trichophrya rotunda* respectively, and these two genera were considered as synonyms of *Heliophrya* because of the late establishment time.

Up to now, three nominal species of *Heliophrya* are recognized: *H. rotunda*, *H. sinuosa* Rieder, 1936, and *H. minima* (Rieder, 1936) Foissner, 1988.

The original description of *H. sinuosa* provided by Rieder (1936) was very short and simple (Figure 10F). It was named as *H. rotunda* var. *sinuosa*, and Jankowski (1981) treated it as a separated species without giving any reason. The main difference between *H. sinuosa* and *H. rotunda* (Figure 10G) is that the former has a regular wavy outline. Considering that the abundance of food will affect the morphology of suctorians, we deduce that these two species may be synonymous.

*Heliophrya minima* is a small species and only about 35μm in diameter. It is characterized by its unfascicled tentacles (Figure 10E): There are up to 22 single tentacles distributed along the cell margin. According to the above characters, it can be easily distinguished from *H. rotunda*. (Matthes, 1988; Foissner et al., 1995).

### *Dendrosoma radians* Ehrenberg, 1838

*Dendrosoma* was established by Ehrenberg (1838) for the multi-branched species, *D. radians* Ehrenberg, 1838. In the following century, several similar genera were erected, but they were all considered to be synonyms of the genus *Dendrosoma*. For example, Perez (1903) erected genus *Lernaeophrya* due to the presence of numerous short actinophores; *Astrophrya* Awerintzew, 1904 was established because of long actinophores and rather short tentacles; Swarczewsky (1928) reported three genera based on organisms found in Lake Baikal: *Baikalophrya*, *Baikalodendron*, and *Gorgonosoma*, which were characterized by the possession of ramified actinophores or a flattened body sprawled over the substrates. In fact, several authoritative protozoologists have considered all these genera to be synonyms of *Dendrosoma* (Hickson and Wadsworth, 1909; Foissner et al., 1995; Dovgal, 2002; Lynn, 2008).

Apart from *Dendrosoma*, *Dendrosomides* Collin, 1906, also has a branched body and numerous actinophores. The latter, however, belongs to the family Dendrosomididae Jankowski, 1978 and exhibits a reproduction mode of exogenous budding. In terms of morphological characteristics, *Dendrosomides* can be easily distinguished from *Dendrosoma* (Figure 10M) by the possession of a stalk; that is, the branched body of *Dendrosomides* is connected to the substrates by a real stalk (Foissner et al., 1995; Figure 10L).

There are only two nominal species in the genus *Dendrosoma*, *D. radians* and *D. capitata* (Perez, 1903) Dovgal, 2002. *D. capitata* was originally reported as *Lernaeophrya capitata* by Perez (1903), and Dovgal combined it into *Dendrosoma* and regarded *Lernaeophrya* as a synonym of *Dendrosoma*. In fact, this species differs from *D. radians* mainly in the number of actinophores (more than 12 vs. about 10 in *D. radians*). Considering that the number of branches and actinophores of *D. radians* are highly variable, however, we deduce that *D. capitata* is a synonym of *D. radians*.

### Phylogenetic Analyses

#### Phylogenetic Position of Four Species and Phylogenetic Relationships in Suctoria

In the ciliate classification system, Suctoria is a special subclass in the phylum Ciliophora. Unlike other ciliates, the suctorians are characterized by a lack of cilia in the trophont, dense cilia covering the body in swarmers, and a polymorphic life cycle. In the system sorted by Corliss (1979), the subclass Suctoria was divided into three suborders: Endogenina Collin, 1912), Exogenina Collin, 1912, and Evaginogenina Jankowski, 1979. This classification system has been widely accepted by subsequent researchers. The Suctoria was divided into four subclasses in the classification system provided by Dovgal (2002): Evaginogenia Jankowski, 1978, Endogenia Collin, 1912, Vermigenia Jankowski, 1978, and Exogenia Collin, 1912. Apart from the three germination modes mentioned in Corliss (1979) (evaginative, endogenous, and exogenous), the fourth mode suggested by Jankowski (1978) was discussed in detail for the first time, namely, vermigemmy. The swarmers of Vermigenia are devoid of ciliature and crawl onto the surface of hosts using a special larval adhesive organelle (tentacle). In view of the lack of cilia in their swarmers, Jankowski established the fourth subclass, Vermigenia. However, the budding modes of these organisms are same as subclass Exogenia, and Dovgal deduced that Vermigemmins should be derived from exogenous ancestors. Lynn (2008) did not accept the fourth subclass and prefer to use the traditional three-group system. We will continue to follow Lynn (2008) classification system until there is new sufficient morphological and molecular evidence of vermigemmy species.

There are few phylogenetic studies on Suctoria due to the lack of molecular information (Snoeyenbos-West et al., 2004). A recent molecular phylogenetic study indicated that the three suctorian orders were divided into three distinct clusters, which is relatively consistent with the classification based on the three reproductive modes of budding (Zhao et al., 2014). In our present research, however, Evaginogenida and Exogenida clustered together and then form sister branches with Endogenida. This clustering also has a good correlation with the budding mode, that is, endogenous budding starts with an invaginated part of the cortex, the brood pouch, and free moving swarmers are formed in the mother cell. Exogenous budding, meanwhile, takes place essentially on the surface of the trophont, and the swarmer pinches off the surface of the mother cell directly to the environment. Evaginative budding involves the formation of a temporary brood pouch, and the swarmer is not freed within the mother cell, but the entire wall of the pouch evaginates out to the cell surface; thus, the process of cytokinesis is similar to exogenous budding (Dovgal, 2002; Lynn, 2008). In other words, exogenous and evaginative budding are ultimately formed on the surface of mother cells, and the free moving swarmer detaches off directly to the environment. Our present phylogenetic tree therefore more reasonably reveals the genetic relationships of the three orders, although the confidence value is low (54% ML, 0.86 BI).

The present study provides the SSU rDNA sequences of four genera/species. Three of these are reported for the first time, namely, *D. radians, Heliophrya rotunda*, and *Peridiscophrya florea*. In the phylogenetic tree, *D. radians*, *C. magna*, and *P. florea* fall in the clade as expected. The position of *H. rotunda* is controversial to Lynn (2008); that is, it falls within Endogenida instead of Evaginogenida. Although *H. rotunda* has been reinvestigated several times, the mode of reproduction had never been mentioned until Matthes (1954) reported its endogenous reproduction. Corliss (1979) established Heliophryidae for this genus, but classified it into Evaginogenida. Mogensen and Butler (1982) also observed that the reproduction mode of *H. rotunda* was endogenous, instead of evaginative. Jankowski (2007) transferred Heliophryidae from Evaginogenia to Endogenia. Based on the above discussion, the family Heliophryidae and genus *Heliophrya* should be transferred to Endogenida.

Until now, there has been no report of the phylogenetic relationship within suctorian order taxa due to the lack of sequence information. The new sequences presented here, however, mean that there are now six genera with sequence information in Endogenida, which gives us the opportunity to explore the family/genus-level relationships. In the current phylogenetic tree, endogenid ciliates are divided into two distinct clusters which are completely consistent with whether or not they have lorica, but the other characters are not well represented. It is impossible to extend the discussion to the relationships within Evaginogenida and Exogenida, however, due to the continuing lack of related sequences.

## Data Availability Statement

The data presented in the study are deposited in the NCBI database repository, accession number(s) MZ912680-MZ912683.

## Author Contributions

MM carried out the experiments, phylogenetic analyses, and drafted the manuscript. YL, QY, and XZ helped to collect samples and performed some experiments. JH, HM, and KA-R helped to write the manuscript, and XC conceived and designed the paper. All authors read and approved the final manuscript.

## Funding

This work was supported by the National Key Research and Development Program of China (2018YFD0900701), the National Natural Science Foundation of China (31970398 and 32070432), the Youth Innovation Promotion Association of the Chinese Academy of Sciences (2019333), and the Research Supporting Project (RSP-2021/10) of the King Saud University, Saudi Arabia.

## Conflict of Interest

The authors declare that the research was conducted in the absence of any commercial or financial relationships that could be construed as a potential conflict of interest.

## Publisher’s Note

All claims expressed in this article are solely those of the authors and do not necessarily represent those of their affiliated organizations, or those of the publisher, the editors and the reviewers. Any product that may be evaluated in this article, or claim that may be made by its manufacturer, is not guaranteed or endorsed by the publisher.

## Footnotes

^1^ http://www.phylo.org/portal2
